# New robust and efficient liquid membranes for conductive vial electromembrane extraction of acids with low to moderate hydrophilicity in human plasma

**DOI:** 10.1007/s00216-024-05503-6

**Published:** 2024-08-29

**Authors:** Chenchen Song, Samira Dowlatshah, Somayeh Gaznawi, Anne Oldeide Hay, Grete Hasvold, Frederik André Hansen

**Affiliations:** 1 Department of Pharmacy, University of Oslo, Blindern, P.O. Box 1068, 0316 Oslo, Norway; 2 School of Chemistry and Chemical Engineering, Henan University of Technology, Zhengzhou, 450001 China

**Keywords:** Sample preparation, Microextraction, Electromembrane extraction, Acidic analytes, New liquid membranes

## Abstract

The current paper reports two new, robust, and efficient conditions for electromembrane extraction of acidic substances from human plasma. Two systems were developed based on eutectic solvents: A1 (“A” for acid) comprised dodecyl methyl sulfoxide and thymol in 1:2 ratio (w/w) as liquid membrane, while A2 used [6-methylcoumarin:thymol (1:2)]:2-nitrophenyl octyl ether in 2:1 ratio (w/w). The performance of A1 and A2 was characterized by extraction of 31 acidic model analytes (pharmaceutical drugs and nutrients) spiked into 100 µL human plasma diluted 1:1 (v/v) with phosphate buffer pH 7.4. The acceptor solution was 50 mM NH_4_HCO_3_ buffer pH 10.0, and extraction was performed at an agitation rate of 750 RPM. Voltage and extraction time were 30 V for 30 min and 10 V for 20 min for A1 and A2, respectively. Under optimal conditions, A1 extracted analytes with 1.8 ≤ log *P* ≤ 6.0 with an average recovery (*R*) of 85.1%, while A2 extracted in a range of 0.5 ≤ log *P* ≤ 6.0 with an average recovery of 79.9%. Meanwhile, extraction current was low at 9 and 26 µA, respectively, which is indicative of good system robustness. Using UHPLC-MS/MS analysis of the acceptor solution, repeatability of the A1 and A2 methods was determined to be 2.8–7.7% and 3.3–9.4% for *R* > 40%, matrix effects were 82–117% and 84–112%, respectively, and linear calibration curves were obtained. The performance and compatibility with human plasma represent a major improvement over previous state-of-the-art liquid membranes for acidic analytes, namely 1-octanol.

**Graphical Abstract:**

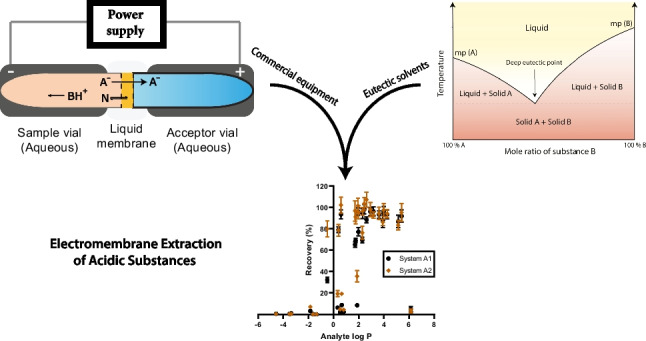

**Supplementary Information:**

The online version contains supplementary material available at 10.1007/s00216-024-05503-6.

## Introduction

During recent decades, microextraction has been a central theme within analytical-scale sample preparation research. Resulting from this, various techniques have been invented and research is still very active. One such technique is electromembrane extraction (EME), first presented in 2006 [1], which mainly has been used for bioanalytical and environmental extraction applications [2]. EME is performed using a supported liquid membrane that separates the sample from a clean acceptor solution. The liquid membrane comprises a porous polymeric membrane, often polypropylene, and a few microliters of organic solvent immobilized in the pores of the membrane by strong capillary forces. Extraction is initiated by applying an external electric field across the liquid membrane, which stimulates charged analyte ions to migrate from the sample, through the liquid membrane, and to the acceptor solution. The latter may subsequently be collected and analyzed by an appropriate analytical method, for example liquid chromatography-mass spectrometry (LC–MS). The organic solvents used in EME are typically non-volatile, non-toxic, and thus safe for the operator. Combined with the very small volume of solvent used, this makes EME a very environmentally friendly technique. EME further offers good selectivity and clean-up of matrix components. Selectivity is controlled by both the polarity and magnitude of the applied electric field, as well as the chemistry and hydrophobicity of the solvent. As such, EME is suitable for extraction of charged analytes across a wide range of properties and hydrophobicity.

To date, approximately 450 articles on EME have been published, exploring fundamentals, methods, and applications, which are summarized in recent reviews [2–6]. Example applications include extraction of metals and heavy metals [7, 8], peptides [9, 10], pharmaceuticals [11, 12], and endogenous metabolites [13, 14]. This has been realized in different technical formats, such as hollow-fiber [15, 16], 96-well [17], and various microfluidic formats [4, 18], all of which have been laboratory-built, and transfer and reproducibility of methods between laboratories have thus been challenging. In 2022, the first commercial EME equipment was however launched, based on using conductive vials to house the sample and acceptor solution [19]. Since then, this vial-based format has been applied for EME in therapeutic drug monitoring (TDM) [12, 20] and analysis of drugs-of-abuse [21, 22].

Recently, we introduced the concept of *generic* EME conditions [23], designed to streamline method development by providing starting conditions for efficient extraction of analytes within a defined range (termed *extraction window*) of charge (*z*) and hydrophobicity (log *P*). A key goal of generic conditions is to lower the barrier to EME’s adoption and to provide both novice and seasoned users with standardized conditions compatible with commercially available equipment. Developing generic systems primarily concerns the selection of a suitable liquid membrane. For example, 2-nitrophenyl octyl ether (NPOE), a much used solvent in EME, has an extraction window of 2.2 ≤ log *P* ≤ 6.4 for + 1 ≤ *z* ≤  + 2 [23]. The establishment of generic conditions is also tied to the operational stability, which mainly concerns the extraction current produced during transfer of charged species. As such, a low current (< 50 µA [24]) minimizes the effects of water electrolysis at the electrodes, which otherwise induces gas formation and pH changes and results in poor extraction efficiency. High current may arise from excessive conductivity of the liquid membrane, a too water-soluble solvent that leaks during the extraction, or poor discrimination of matrix components.

The current fundamental article is the continuation of a series on generic EME systems aimed at commercial conductive vial–based equipment and focuses on acidic compounds displaying low-to-moderate hydrophilicity. Previous papers in this series have concerned generic systems for basic substances of high and low hydrophilicity, in human plasma samples [23, 25, 26]. Acidic substances are however more challenging to extract with EME, for several reasons. Firstly, far fewer suitable liquid membrane solvents have been published, with the vast majority being aliphatic alcohols such as 1-octanol [27]. Alcoholic liquid membranes work by acting as a hydrogen bond donor (HBD) that interacts with the strong hydrogen bonding accepting (HBA) properties of ionized acids, thereby improving partitioning into the organic phase. Compared to solvents for basic analytes, the alcohols however lack secondary interactions such as the cation-π interactions found between cationic analytes and aromatic solvents. This means that solvents used for extracting acids should not be overly hydrophobic since this impedes the transfer of the analyte. Conversely, solvents that are more hydrophilic, like 1-octanol, can increase the permeability of the liquid membrane. This heightened permeability however also allows more competing matrix ions to pass through, which can lead to decreased recovery of the analyte and generate excessive extraction current. Therefore, when using 1-octanol with complex samples, it is often necessary to substantially dilute the samples before proceeding with the extraction process [28]. In this work, we however present two new liquid membranes for acidic analytes based on stronger and more diverse chemical interactions. Compared to 1-octanol, these provided major improvements in the extraction performance and compatibility with human plasma samples without excessive dilution. The liquid membranes were identified after exploring different solvent chemistries and other EME method parameters were subsequently optimized. Thirty-one acidic model analytes were used to characterize the merits of the methods, which had broad extraction windows and high efficiency within, and therefore can be recommended for acids with low to moderate hydrophilicity. It should be emphasized that while LC–MS was utilized for the characterization of the proposed EME methods, their utility is not confined to integration with LC–MS alone. Instead, the work sets the stage for development of new and robust EME applications for acidic analytes.

## Experimental section

### Chemicals and reagents

Camphor, thymol, dl-menthol, dodecyl methyl sulfoxide (DDMS), 6-methylcoumarin, tri(butyl) phosphate, tri(pentyl) phosphate, 1-octanol, 1-nonanol, 1-decanol, 1-undecanol, 2-nitrophenyl octyl ether (NPOE), carvacrol, tri-n-butyl phosphine oxide, tri(isobutyl) phosphate, tris(2-butoxyethyl) phosphate, formic acid (LC–MS grade), dimethyl sulfoxide (DMSO), 4-nitroaniline, and all model analytes (Table S1) were purchased from Sigma-Aldrich (St. Louis, MO, USA). Sodium dihydrogen phosphate dihydrate, disodium hydrogen phosphate dihydrate, ammonium hydroxide, ammonium bicarbonate, and acetonitrile (LC–MS grade) were obtained from VWR (Radnor, PA, USA). Nile red (99%, ACROS Organics™) was purchased from Fisher Scientific (Pittsburgh, PA, USA), and N,N-diethyl-4-nitroaniline was purchased from Fluorochem (Derbyshire, UK). Ultrapure water was produced by a Millipak (0.22 μm filter) Milli-Q water purification system (Molsheim, France). All chemicals and reagents were of analytical grade unless otherwise noted.

Human plasma pooled from multiple blood donor was obtained from the blood bank of Oslo University Hospital (Oslo, Norway) and stored at − 28 °C until use.

### Preparation of solutions

Thirty-one acidic model analytes were used in this study. Each was individually dissolved in methanol, methanol:water 1:1 v/v, or dimethyl sulfoxide, in a concentration of 1.0–5.6 mg mL^−1^. From these stock solutions, a mixture of all at 5 µg mL^−1^ in water was prepared and stored in aliquots at − 28 °C. This solution was used for spiking samples prior to extraction, at a level of 100 ng mL^−1^. The final concentrations of methanol and dimethyl sulfoxide in the extracted samples were 0.15% and 0.10% v/v, respectively.

Eutectic solvents were prepared by mixing appropriate amounts of individual solid components into a 5-mL Eppendorf tube, typically with a total weight of 2 g. Melting was assisted by heating inside an oven at 60 °C for 15 min, which was followed by a brief vortex-mixing. All solvent mixtures (eutectic or non-eutectic) reported below are defined by their weight ratios. Solvents were stored at room temperature and darkness.

### EME equipment and procedure

Electromembrane extraction was performed using equipment based on conductive vials to house the sample and acceptor solution, provided by Extraction Technologies Norway (Ski, Norway). The equipment is illustrated in Figure S1. Unless otherwise stated, conductive vials with a total inner volume of 600 µL and a working volume of 200–500 µL were used. For EME, a pair of vials—one for the sample and another for the acceptor—was fastened into a custom-designed union that housed the supporting membrane infused with the liquid membrane, together forming a single extraction cell. The 9-mm-diameter supporting membrane comprised polypropylene (PP2E, Extraction Technologies Norway). In this configuration, the conductive vials thus served as the anode and cathode. Up to 10 extraction cells were loaded into a 10-position holder mounted on a horizontal shaker (Extraction Technologies Norway) and covered by a lid with one printed electrode for each vial. The lid was connected to a power supply (model ES 0300–0.45, Delta Elektronika BV Zierkizee, Netherlands) and a multimeter (Fluke287, Everett, WA, USA) to monitor the current during extraction.

In preparation for extraction, sample and acceptor solution were loaded into their respective vials, for each extraction cell. Unless otherwise stated, both volumes were 250 µL. The sample comprised a neat standard (analytes spiked into pure buffer) or spiked human plasma (diluted 1:1 v/v with suitable buffer), while the acceptor was a buffer solution. A polypropylene membrane was placed in the Teflon union and secured by tightly screwing the sample vial into place. Next, 9 µL liquid membrane solvent was pipetted onto the supporting polypropylene membrane and became immobilized in the pores. The acceptor vial was screwed into the union, and the extraction cell was placed in the 10-position holder, the lid was attached, and extraction was initiated by simultaneous application of 750 RPM agitation and extraction voltage (variable). The shaking rate and volumes were chosen based on previous optimization [12, 20]. For extraction of acidic analytes, the positive terminal (anode) was connected to the acceptor vial, while the negative terminal (cathode) was connected to the sample vial. Unless otherwise stated, extraction experiments were performed in triplicate. Following extraction, the acceptor solution was analyzed by UHPLC-MS/MS.

### UHPLC-MS/MS analysis

All acidic analytes were quantified using an Agilent 1290 Infinity II UHPLC system (Agilent Technologies, Santa Clara, CA, USA), consisting of a binary pump, an autosampler, and a heated column compartment (40 °C). The column was an Eclipse Plus C18 column (2.1 mm × 50 mm, 1.8 μm, Agilent Technologies) equipped with a guard column of the same type (5 mm). The mobile phase consisted of 0.5% v/v formic acid in each of (A) 95:5 and (B) 5:95 v/v water:acetonitrile, and elution was performed according to the following gradient. Mobile phase B was kept at 0% from 0.00 to 1.00 min, ramped to 53% from 1.00 to 6.00 min, ramped to 100% from 6.00 to 7.00 min, and kept at 100% until 7.50 min when it was returned to 0% for a final 1.50 min re-equilibration. The flow of mobile phases was 0.4 mL min^−1^ during 0.00–7.00 min, 0.7 mL min^−1^ during 7.00–8.50 min, and 0.4 mL min^−1^ during 8.50–9.00 min. The injection volume was 5 µL.

Mass spectrometric detection was performed using an Agilent 6495C triple quadrupole instrument operated in dynamic MRM mode with a cycle time of 300 ms, resulting in a minimum dwell time of 5.82 ms. Gas temperature was 200 °C, gas flow 14 L min^−1^, nebulizer 40 PSI, sheath gas 250 °C, sheath gas flow 12 L min^−1^, and capillary and nozzle voltage were set to 3000 V and 1500 V, respectively, in both positive and negative ionization modes. The ionization mode for each analyte was chosen based on the best signal-to-noise ratio. Individual MRM transitions and collision energies are provided in Table S1.

### UHPLC-UV analysis

Traces of DDMS, thymol, 6-methylcoumarin, and NPOE in the acceptor solution were quantified using a Dionex UltiMate 3000 RS UHPLC system (Thermo Scientific, San Jose, CA, USA) comprising a pump, an auto-sampler, a temperature-controlled column compartment, and a UV detector. UV detection was used rather than MS due to lack of ionization of NPOE. An Acquity UPLC® HSS T3 column (100 × 2.1 mm ID, 1.8 µm, Waters, Wexford, Ireland) maintained at 60 °C was used. The mobile phase consisted of (A) 95:5 v/v deionized water and acetonitrile with 5 mM phosphoric acid and (B) 5:95 v/v deionized water and acetonitrile with 5 mM phosphoric acid, using a flow of 0.4 mL min^−1^ and gradient elution. The gradient was increased linearly from 20 to 100% B in 0.0–5.0 min, and held at 100% B from 5.0–6.0 min, before returning to 20% B in 0.1 min for a final 4 min of re-equilibration. One microliter was injected. DDMS and NPOE were detected at 210 nm, while thymol and 6-methylcoumarin were detected at 280 nm.

### Kamlet-Taft determination

Kamlet-Taft solvent parameters of selected solvents were determined according to a previously reported method [29], using a UV–Vis spectrophotometer (Evolution 201, Thermo Scientific, Waltham, MA, USA) scanning from 300 to 700 nm at 0.5 nm intervals and quartz cuvettes with 1 mm light path. Alternatively, parameters were taken from a previous report using the same method for determination [30]. The Kamlet-Taft values express the relative strength of different solvent interactions, with 0.0 representing weak/no interactions and 1.0 and higher representing strong interactions.

### Computation of physicochemical properties

Physicochemical properties solubility, log *P*, and ionization were obtained computationally from Chemicalize (https://chemicalize.com, ChemAxon, Budapest, Hungary).

### Calculations

Extraction recovery (*R*) was calculated according to the following equation:

$$R=100\%\times\left(\frac{A_{\mathrm{acceptor},\mathrm{final}}}{A_{\mathrm{postspiked}\;\mathrm{blank}\;\mathrm{matrix}}}\right)$$

${A}_{\text{acceptor},\text{final}}$ is the peak area of an analyte obtained by LC–MS analysis of the acceptor solution after extraction. ${A}_{\text{postspiked blank matrix}}$ is the peak area obtained from the acceptor solution after extraction of a blank matrix sample, spiked with analytes in a concentration equal to 100% recovery. In this way, recoveries were corrected for potential matrix effects during LC–MS analysis. Sample and acceptor volumes were equal and therefore not corrected for. The calculation was performed in this manner since the sample concentration (100 ng mL^−1^) was within the linear range of the UHPLC-MS method for all model analytes.

Matrix effects (ME) were calculated according to the following equation:

$$\mathrm{ME}=100\%\times\left(\frac{A_\text{postspiked blank matrix}}{A_\text{unextracted neat standard}}\right)$$

${A}_{\text{unextracted neat standard}}$ is the analyte peak area of a neat standard of equal concentration to postspiked acceptor solution.

## Results and discussion

### Design and requirements of conditions

The establishment of new EME conditions primarily concerns the development of a suitable liquid membrane, and operational parameters such as sample composition and pH, acceptor pH, extraction voltage, and time. The impact on extraction performance of these parameters was assessed using 31 acidic model analytes covering a wide range of hydrophobicity (− 4.5 ≤ log *P* ≤ 6.1). The model analytes were mainly pharmaceutical drugs and some nutrients.

Initially, the liquid membrane composition was considered and assessed using neat standards and human plasma samples. During initial experiments, the sample was adjusted to pH 7.4 using 50 mM phosphate buffer. At this pH, all model analytes were negatively charged. During EME of acids, a boundary layer with depressed pH is formed at the liquid membrane-acceptor interface, which may impede the mass transfer into the acceptor solution [31]. To overcome this effect, the acceptor solution was adjusted to pH 11 by 50 mM ammonia. Ammonia was selected as a volatile base compatible with electrospray ionization. All analytes were confirmed to not degrade at pH 7.4 and 11 (Figure S2).

The final set of conditions was assessed using four formal acceptance criteria, as defined below:

- Criterion 1: The system should be compatible with human plasma samples diluted 1:1 with buffer, and extraction efficiency should be insensitive to sample complexity.
- Criterion 2: The average extraction current should not exceed 50 μA per sample at optimized extraction conditions with complex samples and should not increase during the extraction.
- Criterion 3: The system should extract acidic analytes in a predictable extraction window, based on charge and log *P*.
- Criterion 4: The final method should provide recoveries > 40% and precision < 15% RSD for the majority of compounds within the proposed extraction window.

Criteria 1–2 address the compatibility and robustness of the system with complex samples of limited dilution. A good system should provide similar extraction efficiency and have a low and stable current regardless of the sample complexity. A 1:1 dilution with buffer was considered acceptable as a pH adjustment often is required. The current limit of 50 µA was set based on previous recommendations [23, 24], and the trend should be stable or decreasing since a rising current may indicate increasing transfer of matrix ions and potential poor robustness.

Criterion 3 addresses the predictability of extraction efficiency for new acidic compounds. This implies that suitable systems should not be selective between analytes that fall within the defined windows of charge and hydrophobicity.

Criterion 4 addresses the absolute recovery and precision within the window. In this respect, higher recoveries are desired but exhaustive extraction is not always achieved. From authors’ experience, recoveries exceeding 40% often yield precision better than 15% and most analytes within the extraction window should thus be above this limit.

### Extraction with 1-octanol and other aliphatic alcohols; the typical liquid membranes for acidic analytes

**Fig. 1:**
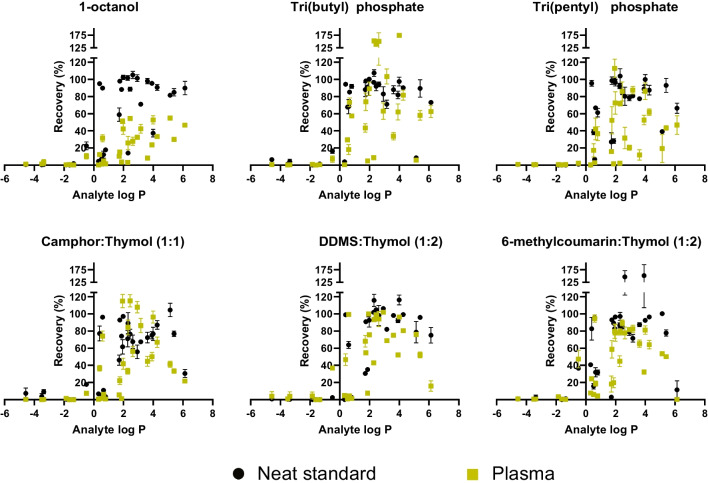
Extraction recovery against analyte hydrophobicity (log *P*) for best candidate liquid membranes identified during screening. Recoveries greater than 100% were attributed to ion enhancement effects in the electrospray ionization process in the UHPLC-MS analysis. Error bars represent the standard deviation (*n* = 3)

In previous literature, 1-octanol and other aliphatic alcohols [27, 32] have been state-of-the-art solvents for EME of acidic analytes. These were therefore investigated in the first set of experiments as a benchmark to compare other solvents. 1-Octanol has a log *P* of 2.58, a water solubility of 0.54 mg mL^−1^, and Kamlet-Taft solvent parameters of *α* = 0.69 (hydrogen bond donor (HBD) properties), *β* = 0.82 (hydrogen bond acceptor (HBA) properties), π = 0.55 (dipolarity/polarizability) [30]. The Kamlet-Taft values express the relative strength of interactions, with 0.0 representing weak/no interactions and 1.0 and higher representing strong interactions. Extractions with 1-octanol as liquid membrane were performed for 30 min at 10 V, with conditions based on initial experiments. Extraction recovery data from neat standard and spiked plasma samples is shown in Fig. 1.

From neat standard samples, 1-octanol had a good performance with low current (10 µA), and the majority of analytes within 1.0 ≤ log *P* ≤ 6.1 were extracted with high efficiency (*R* > 85%). However, for analytes with log *P* < 1.0, partitioning into the liquid membrane was less favorable due to hydrophobic discrimination. In fact, the efficiency drop as log *P* is reduced was very steep due to the logarithmic nature of the log *P* scale. This trend was similar to previous observations for basic substances [23]. The introduction of human plasma destabilized the system, leading to an increase in current, which was attributed to the low hydrophobicity of 1-octanol allowing a greater influx of matrix components, and recoveries were decreased significantly. Replacing 1-octanol with more hydrophobic aliphatic alcohols, namely 1-nonanol, 1-decanol, and 1-undecanol, improved the stability but also decreased recoveries further (data not shown). This was because the hydrophobic barrier of the liquid membrane was increased without any increase in HBA, HBD, or dipole interactions. Despite their frequent use throughout literature, 1-octanol and other aliphatic alcohols were therefore concluded to be unsuitable as generic liquid membranes.

### Screening for alternative liquid membranes

Next, a screening for new alternative liquid membrane solvents was performed. Table S2 summarizes the solvents, their properties, and extraction performance. Depending on the liquid membrane and the current level, 10 V or 30 V was applied for 30 min. Figure 1 shows the extraction efficiency of the best liquid membrane candidates. Recoveries greater than 100% were attributed to ion enhancement effects in the electrospray ionization process in the UHPLC-MS analysis.

First, the stability and performance of 1-octanol with plasma samples were attempted to improve by mixing it 1:1 (weight ratio) with 2-nitrophenyl octyl ether (NPOE), based on previous reports [21, 22, 33]. However, this did not improve the stability. Alternatively, tri(butyl) phosphate was reported by Wu et al. as an efficient liquid membrane for EME of three barbiturates (acids) [34], despite not offering any HBD interactions that otherwise previously was assumed as required for EME of acidic analytes. The Kamlet-Taft values are *α* = 0.00, *β* = 0.88, and π = 0.65, and the solvent is assumed to extract acids primarily by dipole interactions. Tri(butyl) phosphate was highly efficient in extractions from a neat standard sample, but performance deteriorated in plasma samples and current increased with time. Additionally, tri(butyl) phosphate is a suspected carcinogen and was therefore considered an unsuitable solvent. Instead, other phosphates were investigated. Tri(pentyl) phosphate (*α* = 0.00, *β* = 0.80, π = 0.63) yielded a stable and low current (30 µA) and moderate extraction recoveries of analytes of 2 ≤ log *P* ≤ 4. The solvent has recently also been identified as suitable for EME of hydrophobic peptides [30], as well as some basic small-molecule substances [23].

Next, a range of eutectic solvents were considered. (Deep) eutectic solvents are prepared by mixing two (or more) solid components, one HBD and one HBA, that form intermolecular bonds which yield a lower melting point than the individual components. Eutectic solvents are interesting due to the possibilities for fine-tuning solvent properties by mixing various components in different ratios. In EME, monoterpenes, such as menthol and thymol, have been explored as HBD components in eutectic solvents, and applied for peptides [35] and some basic and acidic drugs [29, 36]. Menthol and thymol were paired with three HBA components with complementary properties, camphor (log *P* = 2.5), dodecyl methyl sulfoxide (DDMS, log *P* = 3.6), and 6-methylcoumarin (log *P* = 2.3) in 2:1, 1:1, and 1:2 ratios. Camphor and DDMS are non-aromatic and offer HBA and dipole interaction, while the highly aromatic 6-methylcoumarin offers additional π-type interaction (π-π stacking, anion-π). Menthol-based solvents were all found unsuitable due to either poor solvent stability (did not remain liquid at room temperature), poor extraction efficiency, or dissolution into the acceptor solution. Mixtures with DDMS/6-methylcoumarin and thymol in 2:1 ratio were also not liquid at room temperature. Figure 1 shows the extraction performance of the best ratios for each combination. As seen, DDMS:thymol (1:2) and 6-methylcoumarin:thymol (1:2) were both slightly more efficient than camphor:thymol (1:1), and were therefore considered superior. With DDMS:thymol (1:2), recoveries were also fairly insensitive to a plasma matrix. The Kamlet-Taft values were *α* = 1.11, *β* = 0.30, and π = 0.97, which indicated strong HBD and dipole interactions, and weak-to-moderate HBA interactions. Changing the ratio to 1:1 caused the acceptor solution to become cloudy, which was attributed to the very low solubility of DDMS and consequent formation of microdroplets. For 6-methylcoumarin:thymol, the extraction efficiency was similar for both 1:1 and 1:2 ratios (2:1 was not liquid at room temperature), but the current was reduced from 60 to 30 µA for the 1:2 ratio. The Kamlet-Taft values for this ratio were determined to be *α* = 1.01, *β* < 0.55 (exact value could not be determined due to high background absorbance), and π = 1.09. Based on these observations, DDMS:thymol (1:2) and 6-methylcoumarin:thymol (1:2) were selected as the best candidates. As an additional remark, Hong et al. recently suggested that aromatic liquid membranes may be more susceptible to fouling by matrix components when extracting bases, causing a decrease in extraction recovery [37]. The current data may indicate that this phenomenon is less prominent for acidic analytes, as no major decrease from neat standard to plasma was observed with 6-methylcoumarin:thymol (1:2). We suggest this could be related to the lack of cation-π interactions for the reversed voltage polarity.

A few additional solvents were tested and found unsuitable or inferior; these are summarized in Table S2.

### Optimization

The two candidate liquid membranes, DDMS:thymol (1:2) and 6-methylcoumarin:thymol (1:2), were denoted as systems A1 and A2 (“A” for acid), respectively. As the next step, the final EME conditions for systems A1 and A2 were optimized. First, the final liquid membrane compositions were considered; specifically the addition of NPOE as a stabilizing component for increased robustness was evaluated. For both systems, 1 part NPOE was added to 2 parts of the original solvent. In system A1, addition of NPOE did not give any significant improvement in recovery or current during extraction, while system A2, on the other hand, doubled the recovery for several of the most hydrophobic analytes (Figure S3) and yielded a minor reduction in extraction current. DDMS:thymol (1:2) and [6-methylcoumarin:thymol (1:2)]:NPOE (2:1) were therefore selected as the final liquid membranes for A1 and A2, respectively. The improved recoveries for A2 may have resulted from the relatively weaker interactions provided by NPOE, thereby diminishing the trapping of hydrophobic analytes within the liquid membrane. Kamlet-Taft values for the final A2 mixture could not be determined due to high UV absorbance of NPOE, but are expected to be similar to 6-methylcoumarin:thymol (1:2).

**Fig. 2:**
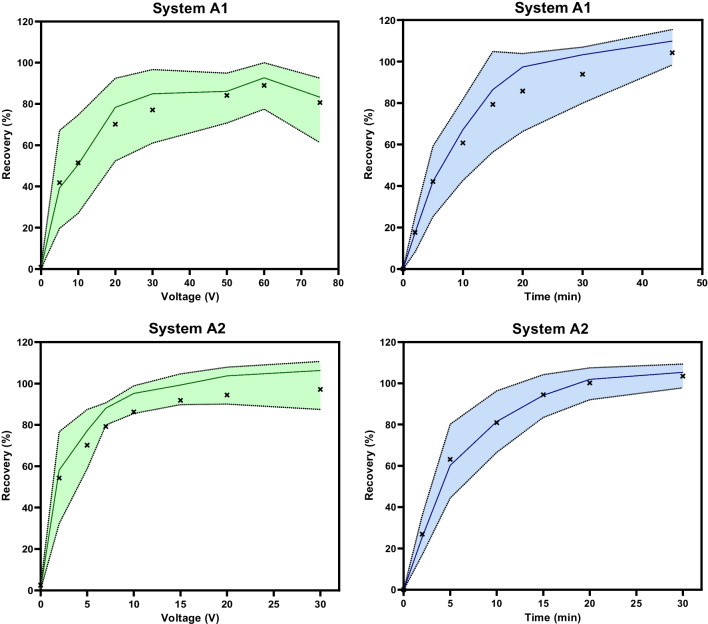
Effect of extraction voltage and time in systems A1 and A2 illustrated as continuous boxplots for analytes with *R* > 40% at any of the tested settings (19 analytes). The solid line represents the median, the colored area is the 25–75% percentile, and points (x) represent the average recovery of all analytes. Voltage was investigated with 20-min extraction time, while extraction time was investigated with 30 V and 10 V for systems A1 and A2, respectively

Next, other extraction parameters were considered, namely liquid membrane volume, sample pH, acceptor pH, agitation rate, extraction voltage, and time. To improve the kinetics of the extraction, smaller sample and acceptor vials (200 µL/100 µL, total capacity/filling volume) were used during optimization. For both A1 and A2, 9–10 µL of liquid membrane was optimal, while smaller volumes gave rise to greater variability between replicates (Figure S4); 9 µL was therefore selected for both methods. The effect of sample pH was investigated for pH 7.4, 9.0, and 11.0. Increasing the sample pH may theoretically improve the extractability for weaker acids; however, all model analytes were already negatively charged at pH 7.4. As such, pH 7.4 was optimal in both systems (Figure S5-6). The decreasing performance at higher pH is explained by electrostatic repulsion of anionic analytes by negatively charged thymol (pKa 10.6) molecules in the sample-liquid membrane interface. Sample pH 7.4 was adjusted by diluting the plasma sample 1:1 (v/v) with 50 mM phosphate buffer (pH 7.4). The acceptor pH was investigated in the range pH 7.0–11.0 (Figure S7-8) and found optimal at pH 10 in both systems. pH 10.0 was therefore selected for the acceptor, which was adjusted using LC–MS compatible (volatile) 50 mM NH_4_HCO_3_ buffer. The difference in optimal sample and acceptor pH is explained by the formation of boundary layers at the water-liquid membrane interfaces that locally have a higher and lower pH than the bulk aqueous solution, at the cathode and anode side of the liquid membrane, respectively [31]. For EME of acids, the acceptor-side boundary layer is thus more acidic and requires a higher acceptor pH to be overcome. Agitation rate was studied at 500–1000 RPM (Figure S9), with 750 RPM found as optimal in both systems. This is concurrent with previous findings in the commercial extraction format [12, 23]. Finally, the impact of extraction voltage and time was investigated. The results are summarized by Fig. 2 as continuous boxplots of recoveries of all analytes extracted with acceptable efficiency (*R* > 40%). Based on these, 30 V and 10 V were selected for systems A1 and A2, respectively, as only minor gains were made at greater voltages. Additionally, greater robustness is expected by operating the systems below the maximum tolerable voltage. Optimal extraction time was set to 30 min and 20 min for systems A1 and A2, respectively, as a compromise between extraction efficiency and throughput. The final optimized parameter settings are provided in Table 1.

**Table 1:** Summary of optimized methods for EME of acidic analytes

	Supporting membrane	Liquid membrane	Sample	Acceptor solution	Agitation	Voltage	Extraction time
A1	PP2E	9 µL DDMS:thymol (1:2)	50 µL plasma + 50 µL phosphate buffer (pH 7.4)	100 µL 50 mM NH_4_HCO_3_ buffer (pH 10.0)	750 RPM	30 V	30 min
A2	PP2E	9 µL [6-methylcoumarin:thymol (1:2)]:NPOE (2:1)	50 µL plasma + 50 µL phosphate buffer (pH 7.4)	100 µL 50 mM NH_4_HCO_3_ buffer (pH 10.0)	750 RPM	10 V	20 min

### Evaluation of systems A1 and A2

**Fig. 3:**
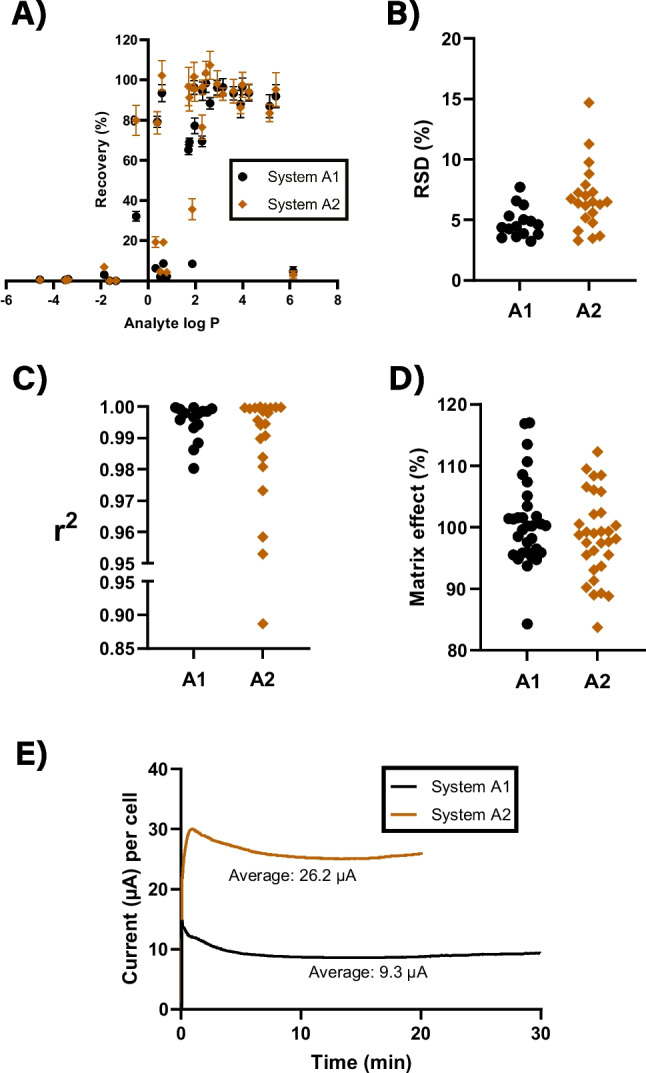
Analytical merits within extraction windows of systems A1 and A2 from human plasma samples. **A** Extraction recoveries as function of analyte hydrophobicity (log *P*). Error bars indicate the standard deviation (*n* = 6). **B** Relative standard deviation (RSD) (*n* = 6). **C** *r*^2^ values for linear calibration curves (5–500 ng mL^−1^) for extracted samples (*n* = 4). **D** Matrix effects for all 31 analytes during LC–MS analysis (average of triplicate). **E** Average current per sample (cell) as function of extraction time

The analytical proficiency of the optimized systems, A1 and A2, was subsequently evaluated. This was not intended as a full validation but aimed to demonstrate their basic analytical merits. The results are summarized in Fig. 3 and additionally provided in Table S2. Both systems extracted all model analytes within 1.0 ≤ log *P* ≤ 5.3 with greater than 40% recovery, except for kynurenic acid (log *P* = 1.87). Additionally, a few more hydrophilic analytes such as enalapril (log *P* = 0.59), piroxicam (log *P* = 0.39), and nicotinic acid (log *P* =  − 0.50) were extracted with high efficiency. For log *P* > 2.3, the extraction efficiency of A1 and A2 was identical. However, system A2 was more efficient for log *P* < 2.3, with the most notable differences observed for kynurenic acid (log *P* = 1.87, *R* = 8.5%/35.7% for A1/A2), furosemide (log *P* = 1.75, *R* = 69.1%/91.2% for A1/A2), 3-indoleacetic acid (log *P* = 1.71, *R* = 65.2%/96.8% for A1/A2), and nicotinic acid (log *P* =  − 0.50, *R* = 32.2%/80.0% for A1/A2). The difference is explained by the lower hydrophobicity of 6-methylcoumarin (log *P* = 2.3) compared to DDMS (log *P* = 3.6). Based on the data in Fig. 3, the extraction windows of systems A1 and A2 were defined as 1.8 ≤ log *P* ≤ 6.0 and 0.5 ≤ log *P* ≤ 6.0, respectively.

For analytes within the extraction windows, the repeatability was 3.2–7.7% and 3.3–14.7% for systems A1 and A2 (Fig. 3B), respectively, and both systems were thus in agreement with criterion 4. Figure 3C shows the linearity of extracted calibration curves, with *r*^2^ > 0.99 for most compounds within the extraction windows. Some compounds were exempted from this, in particular in system A2, but these were mainly with *R* < 40%. Matrix effect during LC–MS analysis was studied for all 31 model analytes and covered the entire span of the LC elution gradient. As seen from the distribution plots in Fig. 3D, no major matrix effects were observed in either system, which indicated good clean-up of the plasma matrix. The current during extraction was stable (Fig. 3E) in both systems and below the defined acceptance criterion of 50 µA. System stability was also evaluated by the dissolution of liquid membrane components into the acceptor phase during extraction, by measuring the individual components with LC-UV. The overall loss of liquid membrane to the acceptor phase was 1.069% ± 0.005 and 0.886% ± 0.037 of the total applied amounts for systems A1 and A2, respectively. The contribution from individual components is shown in Figure S10. The absolute concentration of individual liquid membrane components was < 0.96 mg/mL for A1 and < 0.44 mg/mL for A2. This was considered acceptable and clearly did not cause any disturbance in LC–MS signals (no matrix effects) or current instability.

Previously developed generic systems for bases, B1, B2, and B3 [23, 25], have combined extraction windows from − 2.0 ≤ log *P* ≤ 6.4, but are not suitable for extraction of acids. Systems A1 and A2 both, however, feature HBA and dipole interaction that are favorable for EME of bases, and were therefore briefly tested using a previously reported set of 90 bases (− 4.2 ≤ log *P* ≤ 8.1) and associated UHPLC-MS/MS method [23]. For this, plasma samples were adjusted to pH 2.4 using formic acid, while the acceptor was 100 mM formic acid. All other parameters were as indicated in Table 1. The results are summarized in Figure S11. System A1 extracted within a window of 0.8 ≤ log *P* ≤ 4.2 with an average recovery of 74.3%, while the window for system A2 was 0.4 ≤ log *P* ≤ 3.7 with an average recovery of 68.7%. System A2 however also extracted several bases with log *P* < 0 with high efficiency. Systems A1 and A2 may therefore also be alternatives to the previously proposed B1 and B2 systems.

### Recommendations

Systems A1 and A2 both fulfilled all defined criteria and may therefore be recommended as EME systems for acidic analytes, with extraction windows of 1.8 ≤ log *P* ≤ 6.0 and 0.5 ≤ log *P* ≤ 6.0, respectively. Within their respective extraction windows, the average extraction recovery was 85.1% for A1 and 79.9% for A2. It should be noted that the width of the windows and the performance within are based on a limited number of compounds, and the exact values are therefore uncertain. Considering the price, the ~ 10 mg liquid membrane was estimated to cost 0.079 € and 0.024 € for A1 and A2, respectively, which may be considered negligible in the total analysis cost. The analytical merits were very similar in both systems, and the overall loss of liquid membrane to the acceptor phase was also similar. System A1 however operated at almost threefold less current than A2. Based on these considerations, for overlapping extraction windows system A1 is recommended. The recommendation of both systems is limited to substances with an acidity corresponding to pKa ≤ 6.5. For 6.5 < pKa ≤ 9.0, A1 and A2 may be recommended, but the sample and acceptor pH should be re-optimized and dissolution of the liquid membrane should be reevaluated (due to phenol pKa on thymol). No system can currently be recommended for weak acids with pKa > 9.0, but the authors recommend testing tri(pentyl) phosphate as a liquid membrane that remains unionized at elevated pH. Although not recommended as the first choice for bases, A1 and A2 may also be applied within the extraction windows mentioned above.

These recommendations principally apply to conductive vial–based EME, but similar extraction selectivity is expected for other technical formats.

## Conclusion

A new set of liquid membranes and associated experimental conditions (termed A1 and A2) was developed for acidic substances with 0.5 ≤ log *P* ≤ 6.0. The systems were fully compatible with human plasma samples and extracted most analytes within their defined extraction windows exhaustively (*R* > 85%) or near-exhaustively, which represents a major improvement over previous state-of-the-art EME systems for acids. In addition, they can be applied for EME of basic substances as well, although they are not recommended as the first choice. Adding A1 and A2 to previously published first-choice systems, methods for EME in commercial equipment for a wide variety of basic and acidic substances are now available. In forthcoming research, similar conditions will be investigated for acidic substances with log *P* < 0.5. Also, despite the excellent and robust performance of A1 and A2, one limitation to these systems is their formulation of multiple different components in the liquid membrane mixture. Future research may therefore explore simpler liquid membranes that can deliver the same performance.

With the introduction of commercially available EME equipment, the technique has become more accessible to analytical chemists around the world. We hope this work may serve as starting conditions for future applications of robust EME for acidic substances.

## Supplementary Information

Below is the link to the electronic supplementary material.

Supplementary file1 (DOCX 2826 KB)

## Declarations

### Human plasma

Human plasma from anonymized blood donors was obtained from the blood bank of Oslo University Hospital (Oslo, Norway), which according to Norwegian law can be used for method development purposes without specific ethics approval.
