# Supplementary material for: New robust and efficient liquid membranes for conductive vial electromembrane extraction of acids with low to moderate hydrophilicity in human plasma
Source: Anal Bioanal Chem. 2024 Aug 29;417(7):1293–303. doi: 10.1007/s00216-024-05503-6 (PMC11861104; doi:10.1007/s00216-024-05503-6)

*Supporting information for*

**New Robust and Efficient Liquid Membranes for Conductive-vial Electromembrane Extraction of Acids with Low to Moderate Hydrophilicity in Human Plasma**

Chenchen Song^1,2^, Samira Dowlatshah^1^, Somayeh Gaznawi^1^, Anne Oldeide Hay^1^, Grete Hasvold^1^, Frederik André Hansen^1^*

*^1^Department of Pharmacy, University of Oslo, P.O Box 1068 Blindern, 0316 Oslo, Norway*

*^2^School of Chemistry and Chemical Engineering, Henan University of Technology, Zhengzhou 450001, China*

* Corresponding author.

E-mail address [f.a.hansen@farmasi.uio.no](mailto:f.a.hansen@farmasi.uio.no) (Frederik André Hansen)

**Content**

**Figure S1.** Illustration of EME equipment.

**Table S1.** Analytes, supplier, hydrophobicity, ionization, and LC-MS/MS parameters.

**Figure S2.** Analyte stability plots.

**Table S2.** Solvents screened as liquid membrane, their properties, and extraction performance.

**Figure S3.** Comparison of extraction efficiency for liquid membranes A1 and A2, with/without 2-nitrophenyl octyl ether (NPOE).

**Figure S4.** Effect of liquid membrane volume for system A1.

**Figure S5.** Effect of sample pH for system A1.

**Figure S6.** Effect of sample pH for system A2.

**Figure S7.** Effect of acceptor pH for system A1.

**Figure S8.** Effect of acceptor pH for system A2.

**Figure S9.** Effect of agitation rate for system A1.

**Table S3.** Analytical performance metrics for each analyte in systems A1 and A2.

**Figure S10.** Percentage of the liquid membrane components dissolved in the acceptor phase during extraction.

**Figure S11.** Extraction recoveries of system A1 and A2 applied for EME of 90 basic analytes.

**Figure S1**. Equipment for EME. A) Conductive vials, support membrane union, and circular polypropylene membrane. B) Illustration of EME principle. BH^+^, A^-^, and N represent a protonated base, deprotonated acid, and a neutral substance, respectively. C) Assembled EME unit. D) 10-position vial holder and lid with electrode for each vial.


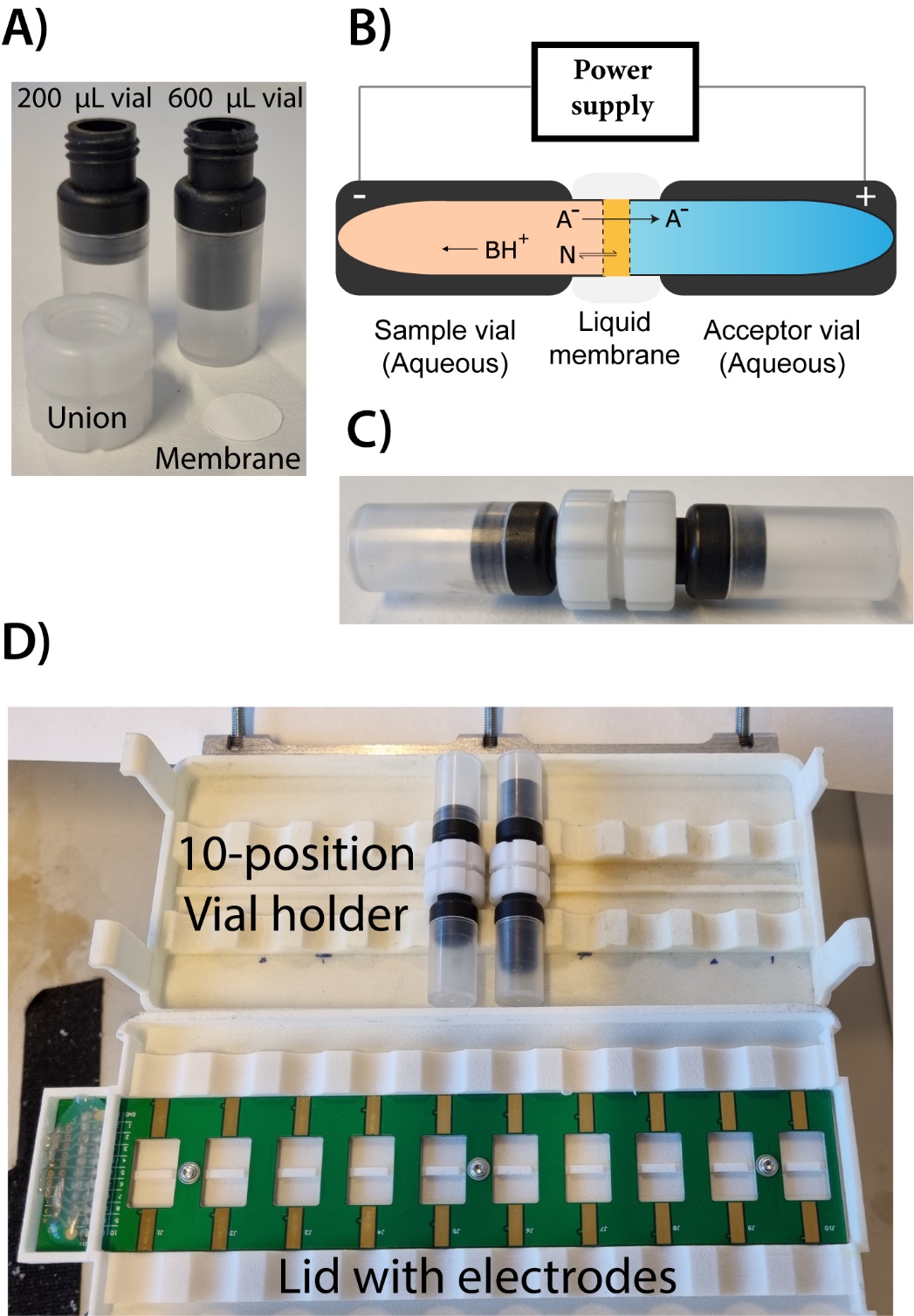


Conductive vial (black) are snap-mounted into a non-conductive bottom (clear) to make them standard HPLC vial height, and provided area for writing sample ID.

**Table S1.** Model analytes and their log P, charge (z), and LC-MS parameters.

| Name | Log P | z_pH 7.4_ | z_pH 10.0_ | z_pH 11.0_ | Retention time (min) | Precursor Ion (m/z) | Product Ion Quantifier (m/z) | Collision energy (V) Quantifier | Product Ion Qualifier (m/z) | Collision energy (V) Qualifier | Ionization mode |
| --- | --- | --- | --- | --- | --- | --- | --- | --- | --- | --- | --- |
| 1-naphthoic acid | 2.62 | -1.0 | -1.0 | -1.0 | 5.3 | 171.0 | 127.1 | 11 |  | 11 | Negative |
| 3-indoleacetic acid | 1.71 | -1.0 | -1.0 | -1.0 | 4.0 | 176.1 | 130.1 | 15 | 103.1 | 30 | Positive |
| Bezafibrate | 3.99 | -1.0 | -1.0 | -1.0 | 6.1 | 362.1 | 316.1 | 11 | 139.0 | 30 | Positive |
| Biotin | 0.32 | -1.0 | -1.0 | -1.0 | 2.9 | 245.1 | 227.1 | 15 | 167.0 | 11 | Positive |
| Bumetanide | 2.30 | -1.0 | -1.7 | -2.0 | 6.2 | 365.1 | 240.1 | 17 | 184.1 | 25 | Positive |
| Chlorpropamide | 1.94 | -1.0 | -1.0 | -1.0 | 5.3 | 277.0 | 111.0 | 38 | 190.0 | 19 | Positive |
| Diclofenac | 4.26 | -1.0 | -1.0 | -1.0 | 6.9 | 296.0 | 250.0 | 11 | 215.0 | 19 | Positive |
| Diflunisal | 3.91 | -1.0 | -1.0 | -1.0 | 6.6 | 251.1 | 233.0 | 11 | 205.1 | 23 | Positive (quant), negative (qual) |
| Doxycycline | -3.37 | -0.5 | -2.4 | -3.0 | 3.9 | 445.2 | 428.2 | 15 | 154.1 | 30 | Positive |
| Enalapril | 0.59 | -1.0 | -1.0 | -1.0 | 4.3 | 377.5 | 117.0 | 41 | 234.1 | 17 | Positive |
| Furosemide | 1.75 | -1.0 | -1.6 | -1.9 | 4.9 | 329.0 | 285.0 | 15 | 205.0 | 23 | Negative |
| Ketoprofen | 3.61 | -1.0 | -1.0 | -1.0 | 5.9 | 255.1 | 209.1 | 15 | 105.0 | 27 | Positive |
| Ketorolac | 2.28 | -1.0 | -1.0 | -1.0 | 5.3 | 256.1 | 105.0 | 19 | 77.1 | 30 | Positive |
| Kynurenic acid | 1.87 | -1.0 | -1.4 | -1.9 | 2.4 | 190.1 | 144.1 | 19 | 116.0 | 30 | Positive |
| L-ascorbic acid | -1.85 | -1.0 | -1.0 | -1.0 | 0.4 | 177.0 | 138.0 | 3 | 99.0 | 11 | Positive |
| Mefenamic acid | 5.40 | -1.0 | -1.0 | -1.0 | 7.3 | 242.0 | 224.0 | 13 | 209.0 | 33 | Positive |
| Methotrexate | -1.63 | -2.0 | -2.0 | -2.0 | 2.7 | 455.0 | 308.0 | 19 |  |  | Positive |
| Nicotinic acid | -0.50 | -1.0 | -1.0 | -1.0 | 0.4 | 124.0 | 53.1 | 30 | 80.0 | 23 | Positive |
| Oxytetracycline | -4.57 | -0.5 | -2.5 | -3.0 | 2.9 | 461.2 | 443.2 | 11 | 426.2 | 19 | Positive |
| Pantothenic acid | -1.36 | -1.0 | -1.0 | -1.0 | 1.1 | 220.1 | 202.1 | 11 | 90.1 | 11 | Positive |
| Piroxicam | 0.39 | -1.0 | -1.0 | -1.2 | 5.1 | 332.1 | 121.1 | 27 | 95.1 | 30 | Positive |
| Probenecid | 2.44 | -1.0 | -1.0 | -1.0 | 6.3 | 284.1 | 140.0 | 30 | 240.2 | 15 | Negative |
| Salicylic acid | 1.98 | -1.0 | -1.0 | -1.0 | 4.1 | 139.0 | 121.0 | 11 | 93.0 | 19 | Positive (quant), negative (qual) |
| Sulfamerazine | 0.52 | -0.7 | -1.0 | -1.0 | 2.5 | 265.1 | 108.0 | 30 | 92.1 | 30 | Positive |
| Sulfamethazine | 0.65 | -0.7 | -1.0 | -1.0 | 2.9 | 279.1 | 92.0 | 33 | 186.0 | 15 | Positive |
| Sulfamethoxazol | 0.79 | -1.0 | -1.0 | -1.0 | 3.7 | 254.1 | 156.0 | 15 | 92.1 | 30 | Positive |
| Sulindac | 2.93 | -1.0 | -1.0 | -1.0 | 5.5 | 357.1 | 340.1 | 23 | 233.1 | 30 | Positive |
| Telmisartan | 6.13 | -1.0 | -1.0 | -1.0 | 4.9 | 258.1 | 305.1 | 9 | 211.0 | 9 | Positive |
| Tetracycline | -3.50 | -0.5 | -2.4 | -3.0 | 3.1 | 445.2 | 410.2 | 19 | 154.1 | 27 | Positive |
| THC-acid (11-Nor-9-carboxy-Δ9-tetrahydrocannabinol) | 5.14 | -1.0 | -1.8 | -2.0 | 7.3 | 345.0 | 327.2 | 17 | 299.3 | 21 | Positive |
| Warfarin | 3.16 | -1.0 | -1.0 | -1.0 | 6.4 | 309.1 | 251.1 | 19 | 163.0 | 11 | Positive |

**Figure S2.** Stability plots for model analytes (100 ng/mL) in 50 mM phosphate buffer (pH 7.4) and 50 mM ammonia (pH 11). Study was performed during 24 hours with signals normalized to signal at t = 0 min.


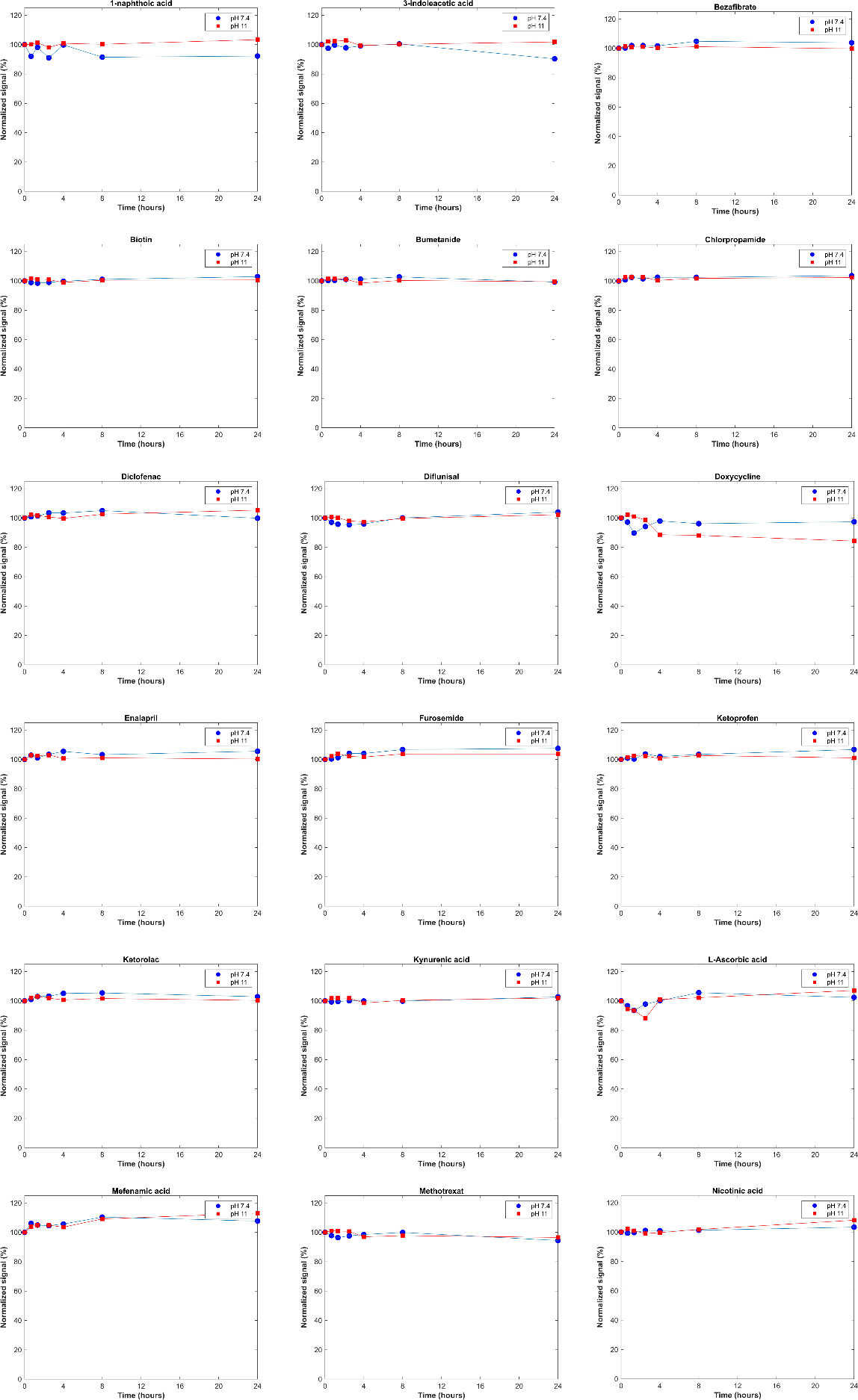


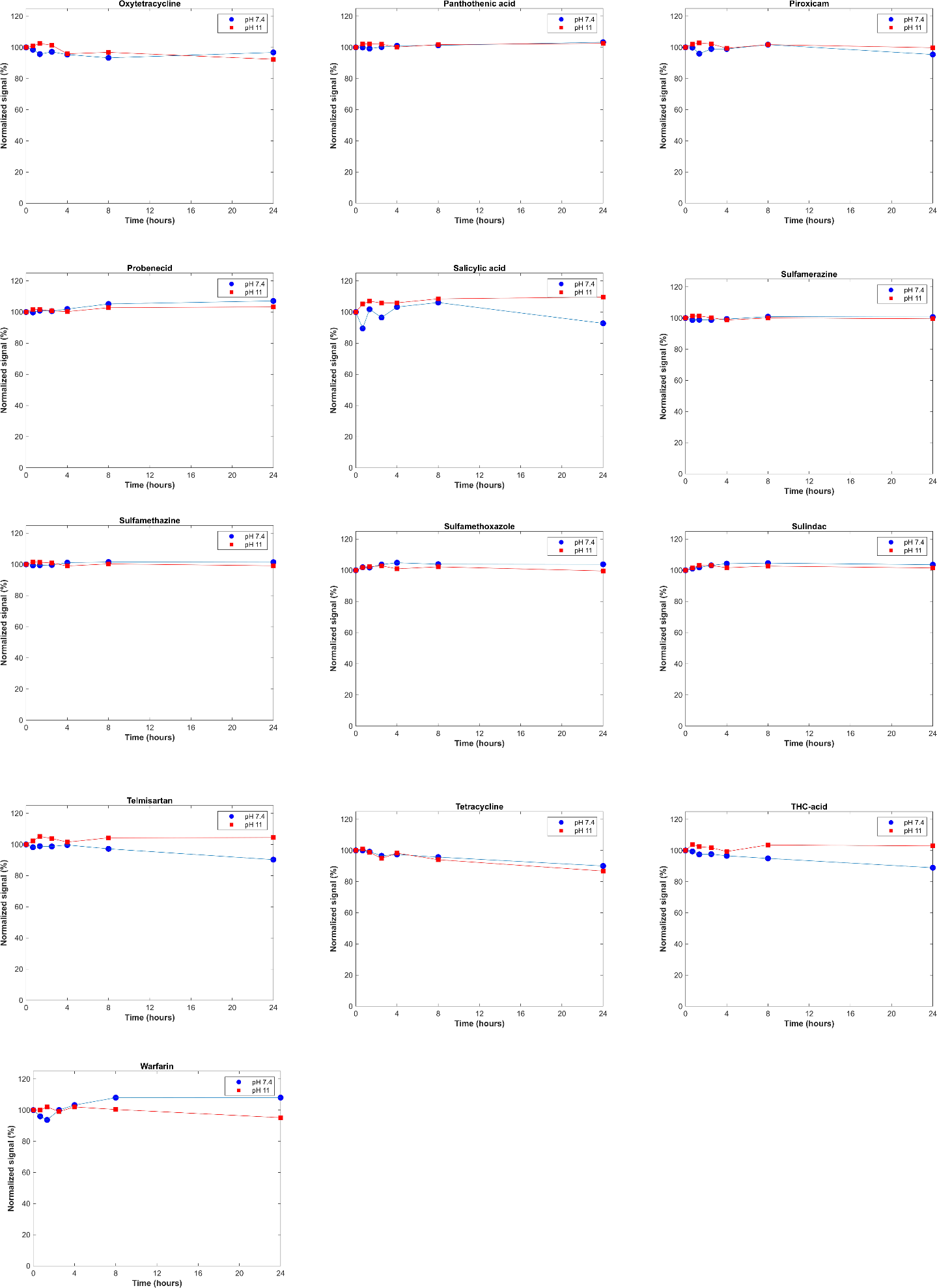


**Table S2.** Comparison of extraction efficiency for liquid membranes A1 and A2, with/without 2-nitrophenyl octyl ether (NPOE). Indicates ratios are w/w. Solvents tested during screening, their physicochemical properties, and performance and suitability as liquid membranes for acidic analytes. The latter is indicated by five categories (A-E): A) Solvent fulfills all acceptance criteria, or solvent unsuitable due to B) high or unstable current in plasma, C) water solubility > 0.5 mg/mL or solvent was dispersed into acceptor solution, D) recoveries are not related to analyte log P and charge, and E) recoveries were poor from plasma samples. DDMS: dodecyl methyl sulfoxide.

| Liquid membrane solvent | HBD sites | HBA sites | Aromatic rings | Log P | Water solubility (mg/mL) | Performance |
| --- | --- | --- | --- | --- | --- | --- |
| *Alcohols* |  |  |  |  |  |  |
| 1-octanol | 1 | 1 | 0 | 2.6 | 0.54 | B, E |
| 1-nonanol | 1 | 1 | 0 | 3.0 | 0.17 | D, E |
| 1-decanol | 1 | 1 | 0 | 3.5 | 0.06 | D, E |
| 1-undecanol | 1 | 1 | 0 | 3.9 | 0.02 | D, E |
| *Phosphates* |  |  |  |  |  |  |
| Tri(butyl) phosphate | 0 | 4 | 0 | 4.1 | 0.03 | B, E |
| Tri(pentyl) phosphate | 0 | 4 | 0 | 5.4 | 0.001 | (E) |
| Tri(isobutyl) phosphate | 0 | 4 | 0 | 3.8 | 0.04 | B, D, E |
| Tris(2-butoxyethyl) phosphate | 0 | 6 | 0 | 3.9 | 0.05 | B, D, E |
| *Other pure solvents* |  |  |  |  |  |  |
| 2-nitrophenyl octyl ether (NPOE) | 0 | 1 | 1 | 4.9 | 0.0008 | D, E |
| Carvacrol | 1 | 1 | 0 | 3.4 | 0.26 | D, E |
| *Solvent mixtures and eutectic solvents (weight ratio)* |  |  |  |  |  |  |
| 1-octanol + NPOE  (2:1)  (1:1) | 1 + 0 | 1 + 1 | 0 + 1 | 2.6 + 4.9 | 0.54 + 0.0008 | B  B |
| 1-octanol + Thymol (1:1) | 1 + 1 | 1 + 1 | 0 + 1 | 2.6 + 3.4 | 0.54 + 0.26 | D, E |
| Tri-n-butyl phosphine oxide + Thymol (1:1) | 0 + 1 | 3 + 1 | 0 + 1 | 2.2 + 3.4 | 0.52 + 0.26 | B, D, E |
| Camphor + Thymol  (2:1)  (1:1)  (1:2) | 0 + 1 | 1 + 1 | 0 + 1 | 2.5 + 3.4 | 1.7 + 0.26 | E  E  E |
| Camphor + Menthol  (1:1) | 0 + 1 | 1 + 1 | 0 | 2.5 + 2.6 | 1.7 + 0.23 | C, D, E |
| Dodecyl methyl sulfoxide (DDMS) + Thymol  (1:1)  (1:2) | 0 + 1 | 1 + 1 | 0 + 1 | 3.6 + 3.4 | 0.003 + 0.26 | C  A |
| Dodecyl methyl sulfoxide (DDMS) + Menthol (1:1) |  |  |  |  |  | D, E |
| 6-methylcoumarin + Thymol  (1:1)  (1:2) | 0 + 1 | 2 + 1 | 2 + 1 | 2.3 + 3.4 | 0.12 + 0.26 | A  A |
| 6-methylcoumarin + Menthol  (1:1)  (1:2) |  |  |  |  |  | Not liquid at room temperature |

**Figure S3.** Comparison of extraction efficiency for liquid membranes A1 and A2, with/without 2-nitrophenyl octyl ether (NPOE). Indicated ratios are w/w. Error bars represent the standard deviation.


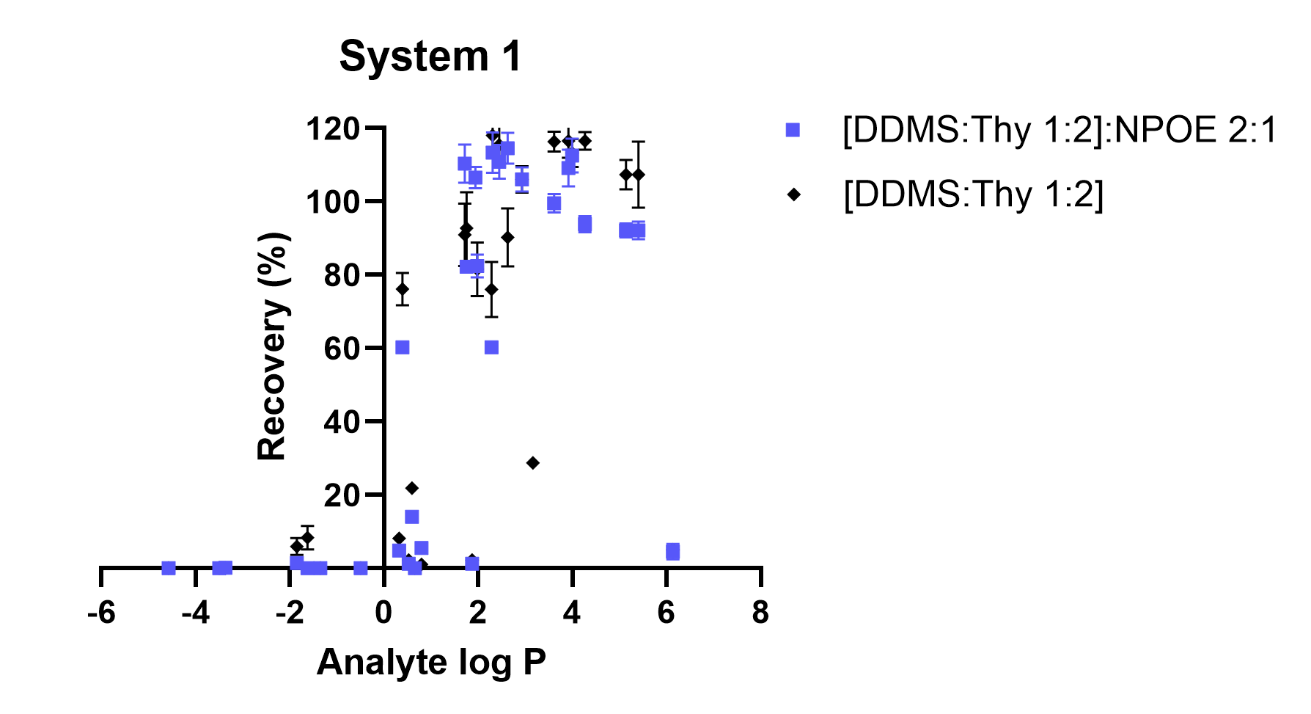

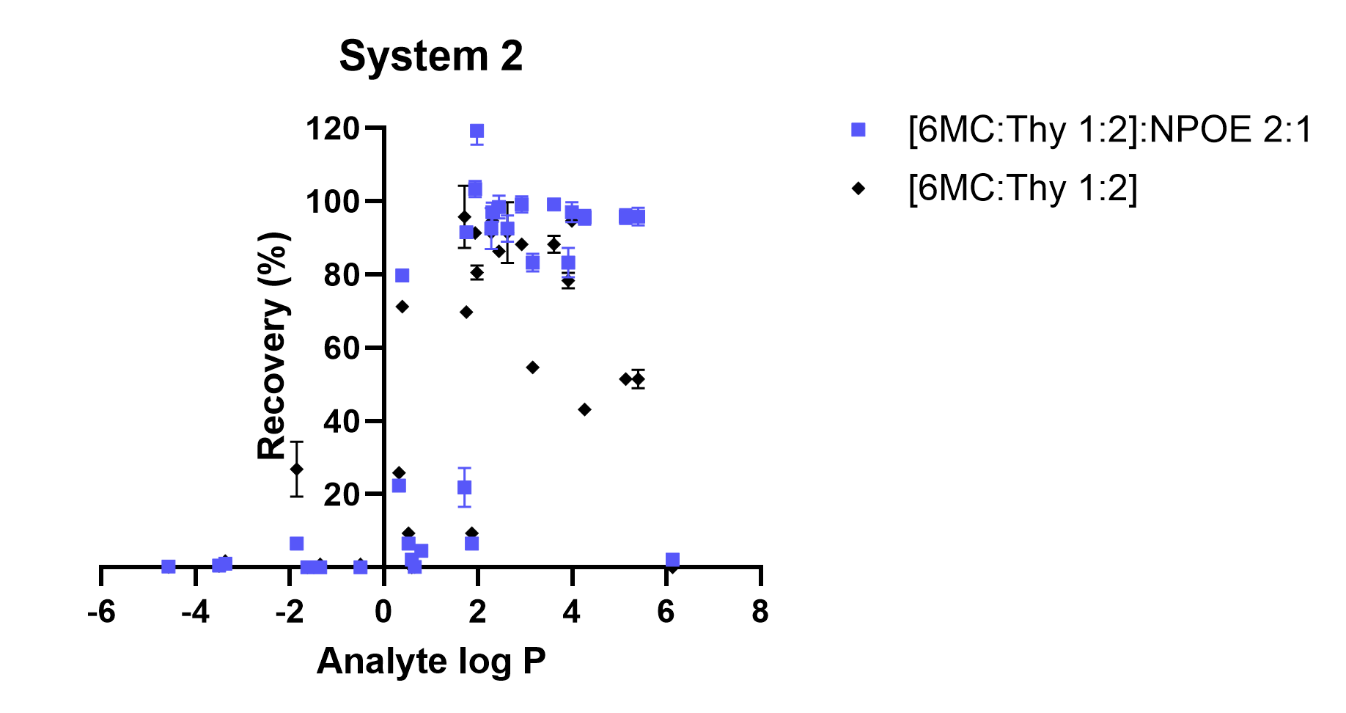


**Figure S4.** Effect of liquid membrane volume for system A1. Error bars represent the standard deviation. Extraction conditions: 30 V, 20 min, 750 RPM, sample pH 7.4 (50% plasma), acceptor pH 10.0.

**
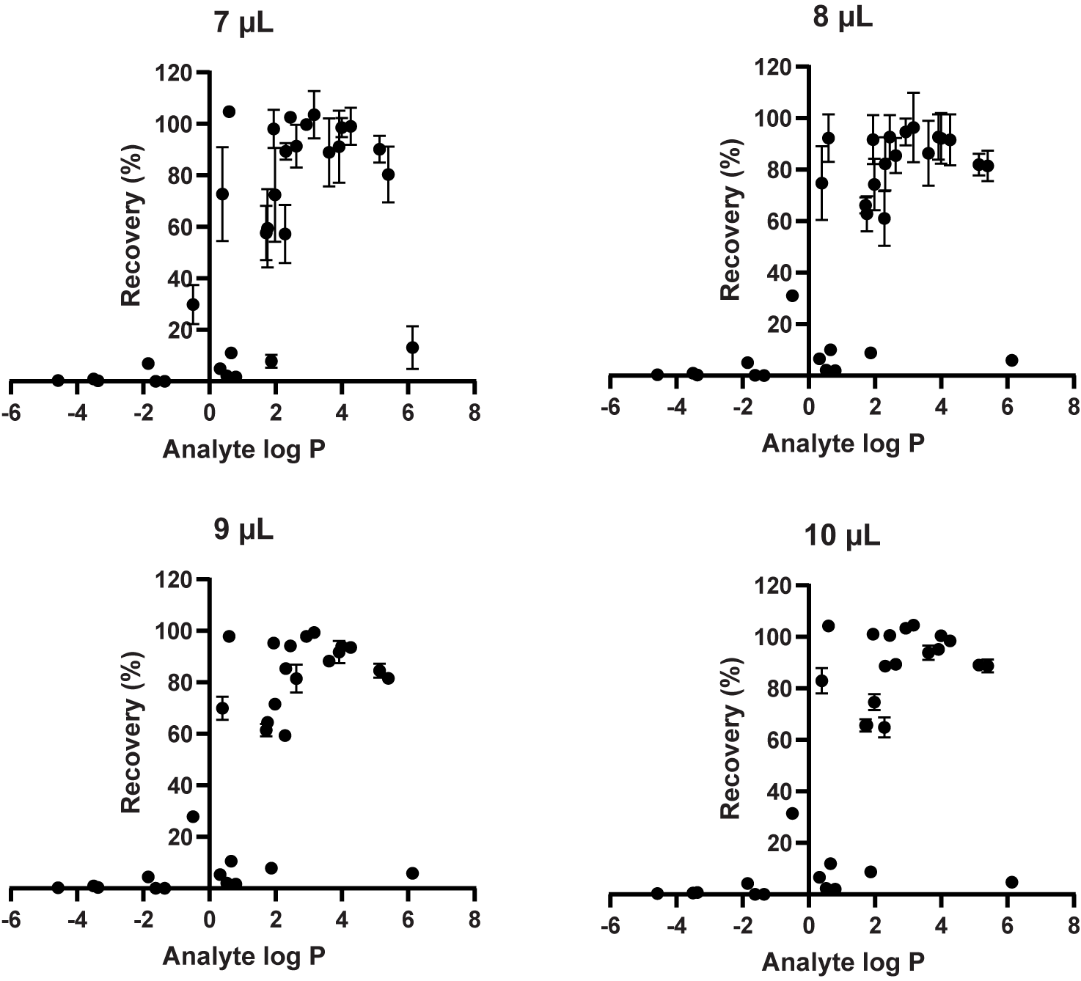
**

**Figure S5.** Effect of sample pH for system A1. Error bars represent the standard deviation. Extraction conditions: 9 µL liquid membrane, 30 V, 20 min, 750 RPM, acceptor pH 10.0.


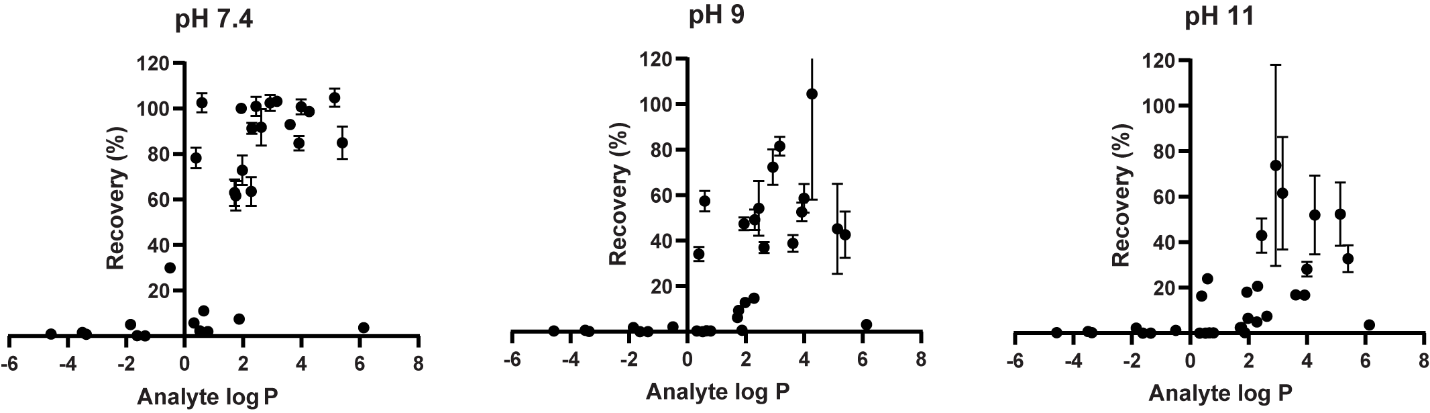


**Figure S6.** Effect of sample pH for system A2. Error bars represent the standard deviation. Extraction conditions: 9 µL liquid membrane, 30 V, 20 min, 750 RPM, acceptor pH 10.0.


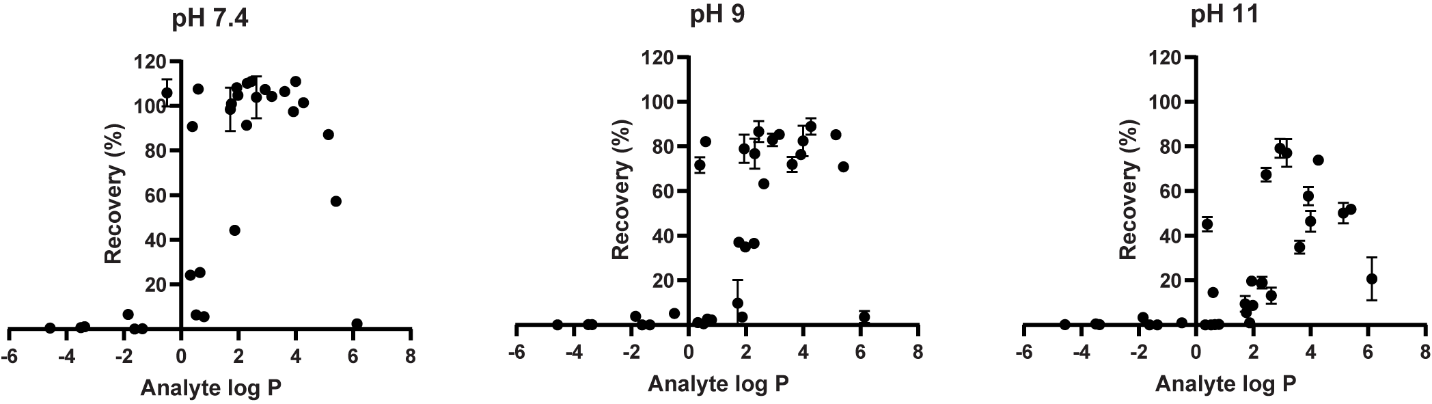


**Figure S7.** Effect of acceptor pH for system A1. Error bars represent the standard deviation. Extraction conditions: 9 µL liquid membrane, 30 V, 20 min, 750 RPM, sample pH 7.4 (50% plasma).


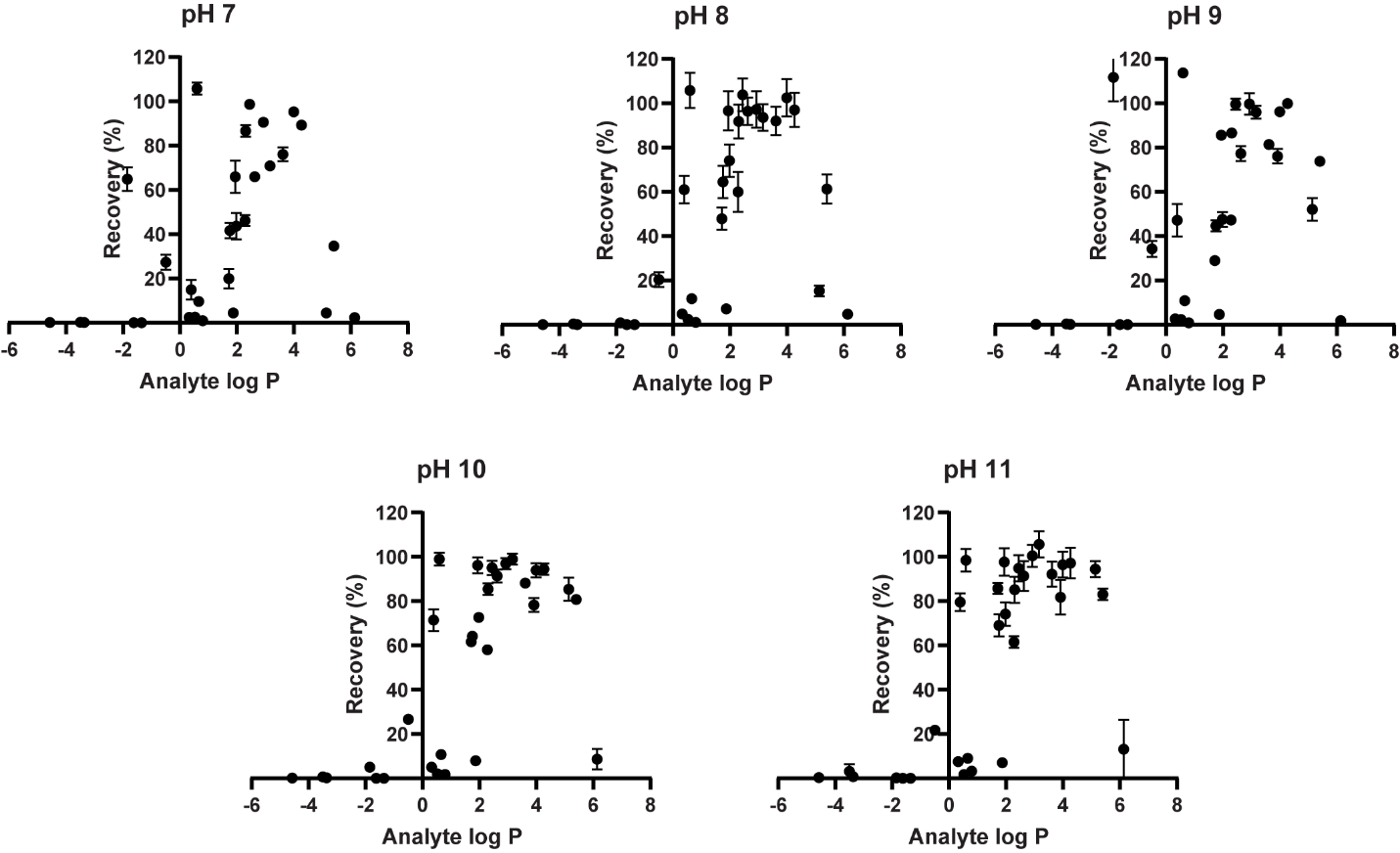


**Figure S8.** Effect of acceptor pH for system A2. Error bars represent the standard deviation. Extraction conditions: 9 µL liquid membrane, 30 V, 20 min, 750 RPM, sample pH 7.4 (50% plasma).


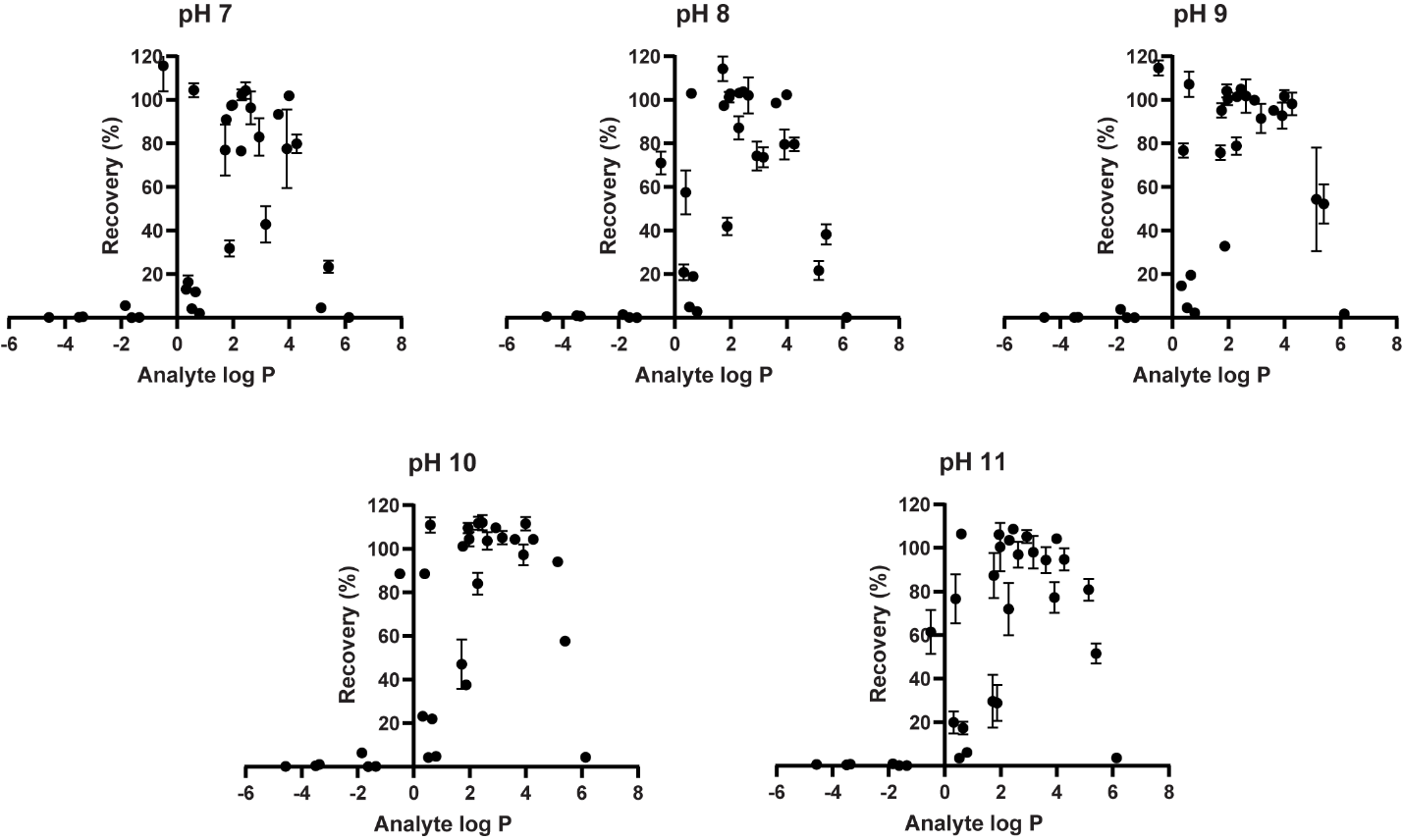


**Figure S9.** Effect of agitation rate for system A1. Error bars represent the standard deviation. Extraction conditions: 9 µL liquid membrane, 50 V, 20 min, sample pH 7.4 (50% plasma), acceptor pH 10.0.


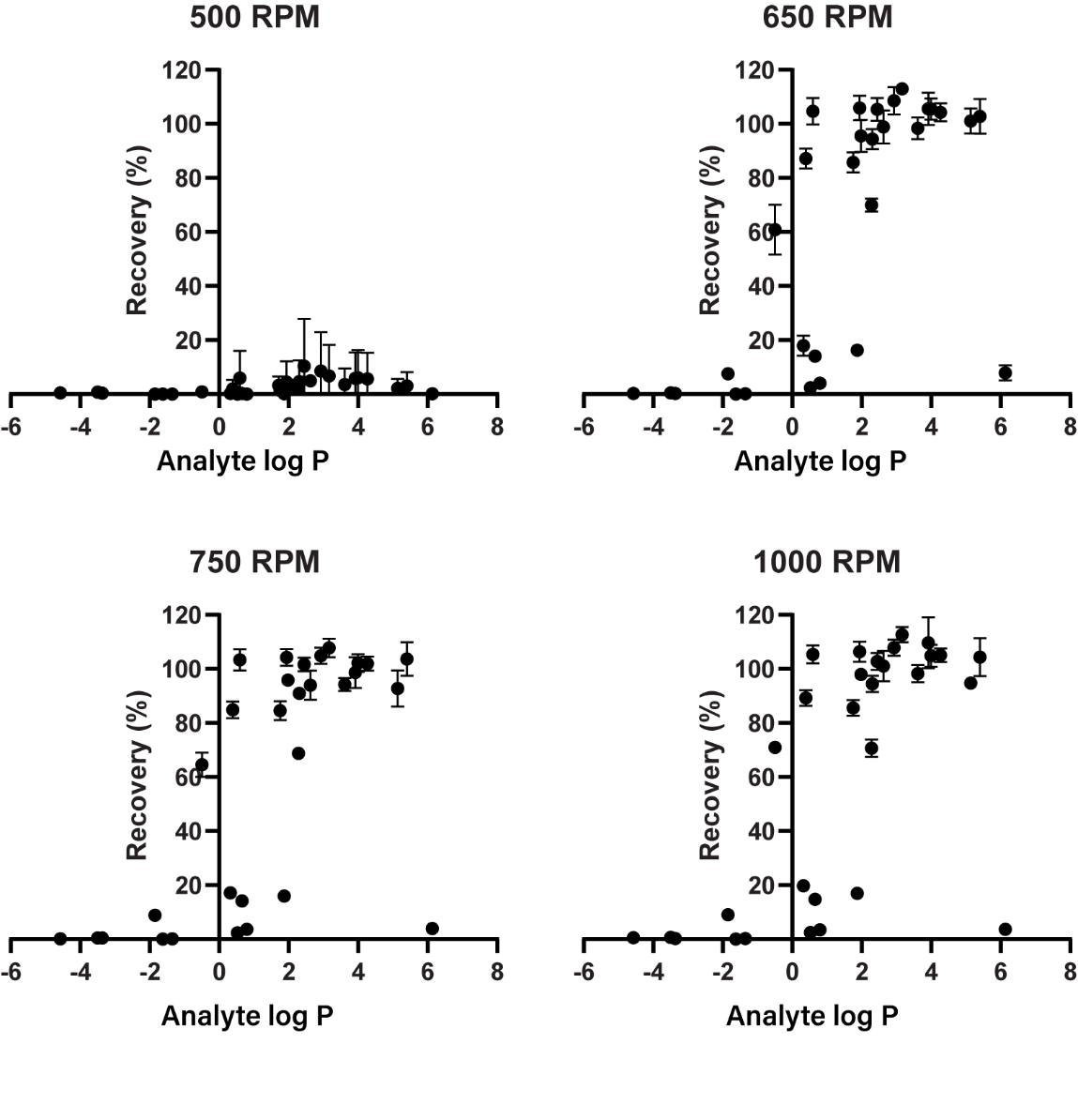


*Optimal agitation rate is generally known to be independent of type of liquid membrane. Optimization was therefore only performed for system A1.

**Table S3.** Extraction recovery, RSD, r^2^ values, linear range, and matrix effects for optimized methods A1 and A2 applied for human plasma samples. Values related to linearity are not included for analytes outside the extraction windows since generally suffer poor recovery and linearity.

| Analyte | A1 recovery (%) | A1 RSD (%) | A2 recovery (%) | A2 RSD (%) | A1 r^2^ | A1 linear range (ng/mL) | A2 r^2^ | A2 linear range (ng/mL) | A1 ME (%) | A1 ME (%) |
| --- | --- | --- | --- | --- | --- | --- | --- | --- | --- | --- |
| 1-naphthoic acid | 88.5 | 3.2 | 107.4 | 6.5 | 0.9958 | 5-500 | 0.9985 | 5-500 | 99 | 98 |
| 3-indoleacetic acid | 65.2 | 3.9 | 96.8 | 9.8 |  |  | 0.8868 | 5-500 | 117 | 89 |
| Bezafibrate | 96.9 | 4.3 | 97.6 | 6.4 | 0.9993 | 5-500 | 0.9996 | 5-500 | 98 | 99 |
| Biotin | 6.3 | 13.8 | 19.4 | 14.4 |  |  |  |  | 100 | 101 |
| Bumetanide | 94.4 | 4.9 | 96.5 | 3.7 | 0.9985 | 5-500 | 0.9999 | 5-500 | 98 | 98 |
| Chlorpropamide | 96.4 | 3.5 | 101.6 | 7.0 | 0.9985 | 5-500 | 0.9998 | 5-500 | 96 | 96 |
| Diclofenac | 93.6 | 4.4 | 94.2 | 4.1 | 0.9979 | 5-500 | 0.9993 | 5-500 | 96 | 99 |
| Diflunisal | 88.1 | 7.7 | 86.2 | 3.5 | 0.9862 | 5-200 | 0.9945 | 5-500 | 84 | 89 |
| Doxycycline | 0.9 | 56.5 | 0.6 | 40.3 |  |  |  |  | 103 | 89 |
| Enalapril | 93.5 | 4.6 | 102.2 | 7.3 |  |  | 0.9996 | 5-500 | 107 | 102 |
| Furosemide | 69.1 | 2.8 | 91.2 | 7.2 |  |  | 0.9907 | 5-500 | 95 | 90 |
| Ketoprofen | 93.4 | 3.6 | 94.5 | 6.3 | 0.9997 | 5-500 | 0.9996 | 5-500 | 96 | 96 |
| Ketorolac | 69.5 | 3.9 | 76.5 | 7.9 | 0.9997 | 5-500 | 0.9956 | 5-500 | 96 | 93 |
| Kynurenic acid | 8.5 | 5.3 | 35.7 | 14.7 | 0.9803 | 5-500 | 0.9732 | 5-500 | 102 | 100 |
| L-Ascorbic acid | 3.2 | 11.0 | 7.0 | 18.2 |  |  |  |  | 111 | 112 |
| Mefenamic acid | 91.9 | 6.2 | 95.2 | 8.8 | 0.9943 | 5-500 | 0.9979 | 5-500 | 95 | 99 |
| Methotrexat | 0.1 | 70.2 | 0.1 | 65.2 |  |  |  |  | 101 | 102 |
| Nicotinic acid | 32.2 | 7.6 | 80.0 | 9.4 |  |  |  |  | 114 | 108 |
| Oxytetracycline | 0.7 | 102.3 | 0.5 | 134.7 |  |  |  |  | 100 | 97 |
| Panthothenic acid | 0.0 | 76.9 | 0.1 | 9.4 |  |  |  |  | 101 | 99 |
| Piroxicam | 79.2 | 3.5 | 78.7 | 7.1 |  |  |  |  | 100 | 84 |
| Probenecid | 98.3 | 4.6 | 103.5 | 5.6 | 0.9978 | 5-500 | 0.9998 | 5-500 | 94 | 91 |
| Salicylic acid | 77.2 | 5.0 | 95.6 | 6.5 | 0.9991 | 5-500 | 0.9838 | 5-500 | 105 | 110 |
| Sulfamerazine | 2.1 | 12.2 | 4.6 | 4.8 |  |  | 0.9584 | 5-500 | 101 | 99 |
| Sulfamethazine | 8.7 | 8.2 | 19.2 | 6.2 |  |  | 0.9529 | 5-500 | 102 | 107 |
| Sulfamethoxazole | 2.3 | 4.1 | 4.3 | 11.3 |  |  | 0.9808 | 5-500 | 100 | 98 |
| Sulindac | 96.2 | 3.8 | 98.1 | 6.8 | 0.9966 | 5-500 | 0.9995 | 5-500 | 96 | 96 |
| Telmisartan | 4.9 | 46.9 | 3.1 | 57.4 |  |  |  |  | 109 | 106 |
| Tetracycline | 0.5 | 61.3 | 0.3 | 74.1 |  |  |  |  | 117 | 108 |
| THC-acid | 87.0 | 6.6 | 83.5 | 5.2 | 0.9932 | 5-500 | 0.9898 | 5-500 | 95 | 94 |
| Warfarin | 96.4 | 4.5 | 93.0 | 3.3 | 0.9884 | 5-500 | 0.9941 | 5-500 | 102 | 106 |

**Figure S10.** Percentage of the liquid membrane components that were dissolved in the acceptor solution during extraction. The residue of components in the acceptor solution was measured by LC-UV and normalized by the signal equal to 100% dissolution. The overall loss was calculated as the average component loss weighted by the relative ratios in the solvent. Error bars represent the standard deviation (n=4).


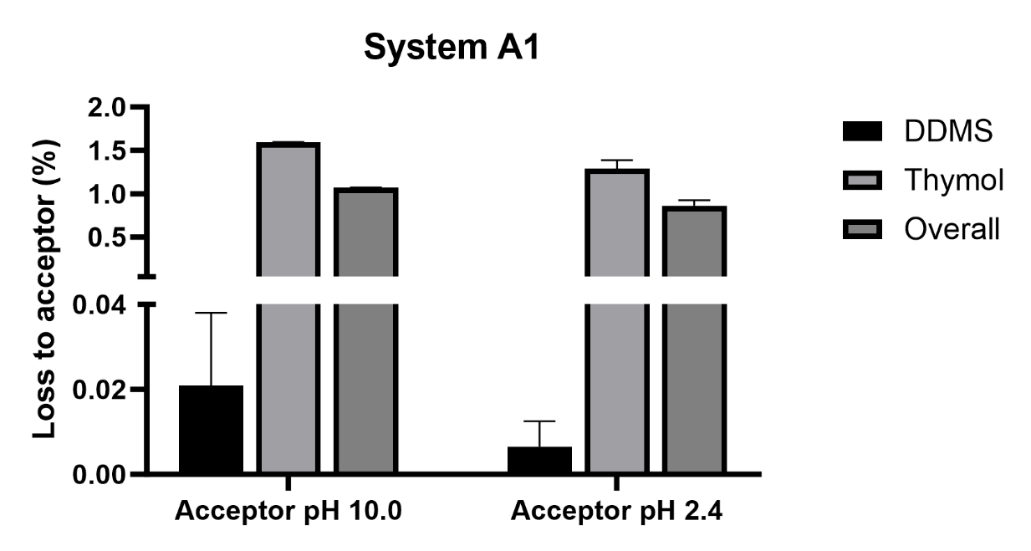

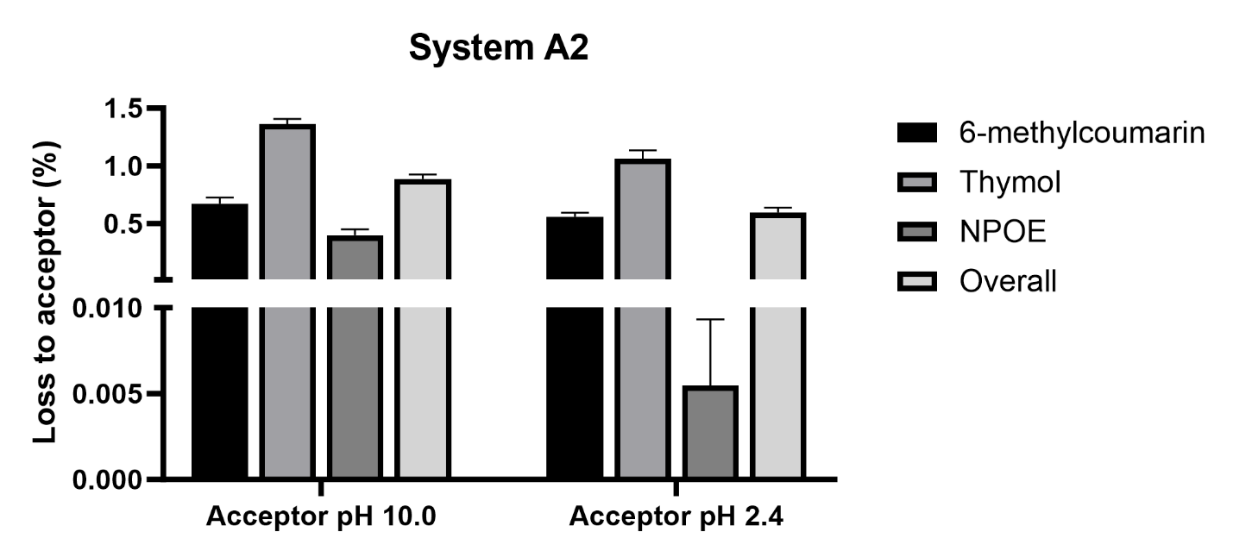


**Figure S11.** Extraction recoveries versus analyte log P value for systems A1 and A2 applied for extraction of 90 basic substances. Below, current profiles for the respective extractions are shown. Sample: human plasma diluted 1:1 (v/v) with 900 mM HCOOH and spiked to 100 ng/mL of each model analyte. Acceptor: 100 mM HCOOH. All other conditions were as reported for A1 and A2. Error bars represent the standard deviation (n=4).


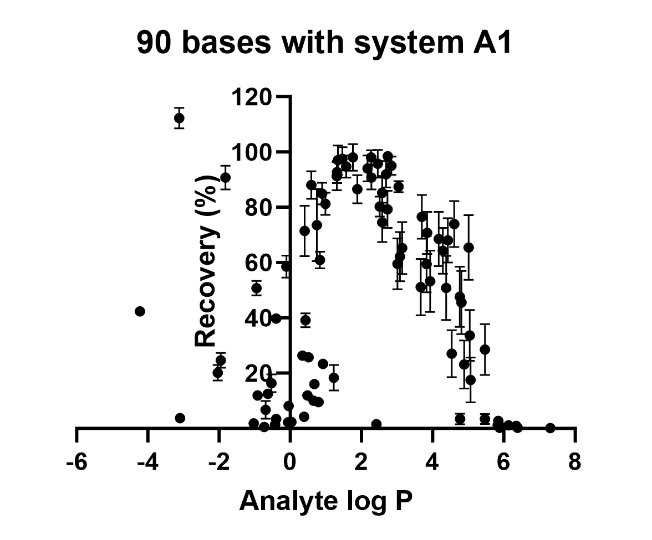

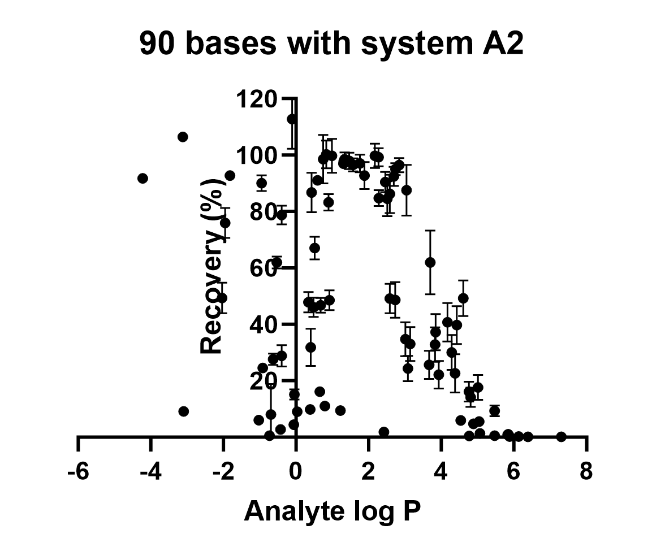

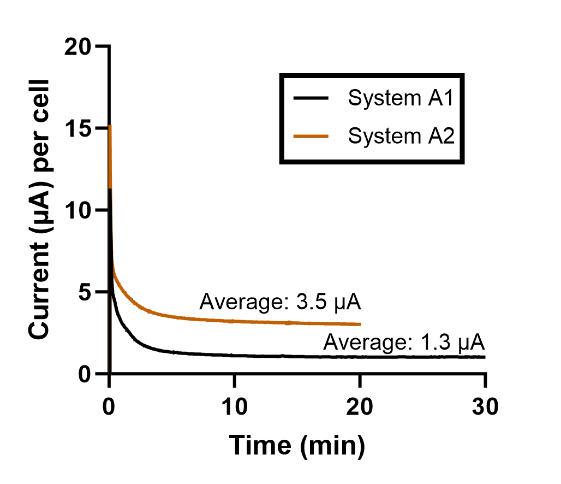

Supplement: Supplementary file 1 — Supplementary file1 (DOCX 2826 KB) [file 216_2024_5503_MOESM1_ESM.docx]
